# Effect of bacterial endotoxin lipopolysaccharide treatment on duck Leydig cells

**DOI:** 10.21451/1984-3143-AR2019-0002

**Published:** 2019-11-18

**Authors:** Yongcong Lao, Hongjia Ouyang, Xuebing Huang, Yunmao Huang

**Affiliations:** 1 Guangdong Province Key Laboratory of Waterfowl Healthy Breeding, Guangzhou, China; 2 Zhongkai University of Agriculture and Engineering, College of Animal Science & Technology, Guangzhou, China

**Keywords:** duck, Leydig cells, LPS, testosterone, gene expression

## Abstract

This study aimed to investigate the effects of bacterial endotoxin lipopolysaccharide (LPS) on hormone production and gene expression in duck Leydig cells and its underlying mechanisms. Leydig cells were collected from 200-day-old mallard ducks and divided into five treatment groups (0, 50, 100, 200, and 400 ng/mL LPS). After treatment with LPS for 6, 12, 24, and 48 h, testosterone, activin, and inhibin levels in the cell supernatants were determined using enzyme-linked immunosorbent assay (ELISA) kits. The expression levels of testosterone synthesis-related genes, including steroidogenic acute regulatory protein (*StAR*), 3-beta-hydroxysteroid dehydrogenase (*3β-HSD*), and cytochrome P450 aromatase (*P450arom*), and reproductive-related genes, including gonadotropin-inhibitory hormone receptor (*GnIHR*), follicle stimulating hormone receptor (*FSHR*), and luteinizing hormone receptor (*LHR*) were detected using quantitative real-time polymerase chain reaction (qRT-PCR). We successfully isolated and cultured duck Leydig cells with cell purity above 90%. Compared with the control group, the levels of testosterone, activin, and inhibin secreted in Leydig cells decreased gradually with increasing LPS concentration. After treatment with LPS, the expression of *StAR* and *3β-HSD* genes in Leydig cells was upregulated at 12 h, and that of *GnIHR* was upregulated at 24 h; whereas the expression of *FSHR* and *LHR* was reduced at 24 h. This study indicates that LPS can inhibit the secretion of hormones and regulate the expression of related genes in duck Leydig cells.

## Introduction

Leydig cells are endocrine cells distributed in the loose connective tissue between the seminiferous tubules of the testicle. They account for about 2%-4% of the total number of testicular cells (Grzywacz et al., 1998; Wang et al., 1999). The main functions of Leydig cells are to synthesize and secrete testosterone (T), and about 95% of testosterone in the body is synthesized by these cells. Leydig cells promote spermatogenesis, the development and differentiation of male reproductive organs, and maintain secondary sexual characteristics and sexual function through the effects of testosterone (Chen et al., 2001).

Testosterone biosynthesis is mainly catalyzed by steroidogenic acute regulatory protein (StAR), hydroxysteroid dehydrogenase (3β-HSD, 17β-HSD), and the cytochrome P450 family (P450c17, P450scc, and P450arom) in response to the gonadotropin, luteinizing hormone (LH), in Leydig cells (Nagata et al., 1999; Topo et al., 2009; Raucci et al., 2014). The presence and development of Leydig cells are related to the secretion of reproductive hormones by the hypothalamic-pituitary-testicular axis, mainly follicle-stimulating hormone (FSH), LH, gonadotropin-releasing hormone (GnRH), and gonadotropin-inhibitory hormone (GnIH). The FSH promotes testicular development, and LH promotes steroid synthesis and differentiation. Furthermore, GnRH promotes FSH and LH to regulate Leydig cells by binding to specific receptors, whereas GnIH regulates Leydig cells by inhibiting GnRH in the hypothalamus or directly inhibiting LH.

Lipopolysaccharide (LPS) is an active component of the cell wall of Gram-negative bacteria; it activates host immune cells via toll-like receptor 4 (TLR-4) signaling (Wu et al., 2017; Anthoney et al., 2018; Morizane et al., 2019). The LPS binds to TLR-4 and activates the NK-κB signaling pathway (Carmody and Chen, 2007; Lee and Kim, 2007), and thus activates the NK-κB downstream signaling pathway to induce pro-inflammatory responses (O’Neill et al., 2013; Vijay, 2018). The pro-inflammatory response adversely affects reproductive function in animals, impairs neuroendocrine function, and disturbs the ovarian cycle (Kajihara et al., 2006; Sharova et al., 2014). Previous studies have shown that after treatment with LPS, testicular damage in the rabbit buck becomes evident, with a reduction in the number of germinal cells and increase in the number of structurally altered Sertoli cells on the seventh day (Brecchia et al., 2010; Collodel et al., 2012). Some studies have demonstrated that LPS inhibits testicular steroidogenesis and spermatogenesis by inducing inflammation (O’Bryan et al., 2000).

In vitro cultured Leydig cells have the ability to secrete basal testosterone, stimulate a response to human chorionic gonadotropin, and can be effectively used to study reproductive function in animals. Various methods for the isolation and culture of Leydig cells from humans, mice, chickens, and many other species have been reported; however, there have been no corresponding reports on ducks. In this study, Leydig cells of mallard ducks were isolated and cultured, and the effects of LPS on Leydig cells and its regulatory mechanisms were studied.

## Methods

All experimental procedures involving animals were conducted in conformity with the guidelines on the care and use of laboratory animals, formulated by the Ministry of Science and Technology of China.

### Isolation and purification of Leydig cells

Leydig cells were isolated from the testis of 200-day-old mallard *Anas platyrhynchos* ducks. Consistent with the methods of previous studies, we used collagenase digestion and Percoll density gradient centrifugation to isolate and purify the Leydig cells (Molenaar et al., 1986; Toocheck et al., 2016; Shi et al., 2017).

Each testis was digested in a 50-mL centrifuge tube within an oscillating incubator (200 rpm and 37 °C) for 30 min by Dulbecco’s modified Eagle medium nutrient mixture F-12 (DMEM/F-12) (Gibco, Carlsbad, CA, USA) containing 0.1% collagenase II. The digestion was stopped by adding an equal volume of DMEM/F-12. The supernatant containing the Leydig cells was separately filtered through a 70-mm nylon cell strainer and a 40-mm nylon cell strainer (Biologix Group Limited, Shandong, China). The filtrate was centrifuged at 1000 rpm for 5 min at 4 °C. To obtain purified Leydig cells, the pellet was resuspended in 5 mL of DMEM/F-12, loaded onto the top of a discontinuous Percoll density gradient (17, 25, 40, and 60%; GE Healthcare Bio-Sciences AB, Uppsala, Sweden), and subsequently centrifuged at 3000 rpm for 30 min at 4 °C (Hedger and Eddy, 1987). The cells in the third layer were collected and washed twice with DMEM/F-12. The purified cells were resuspended in DMEM/F-12 containing 10% fetal bovine serum (FBS) (Gibco, USA), antibiotic-antimycotic (penicillin, 50 IU/mL; streptomycin, 50 mg/mL) (Gibco). The cells were plated at a density of 10^6^ cells/mL in 12-well plates (Corning Costar, USA) at 1 mL/well and maintained at 37 °C with 5% carbon dioxide.

### Cell purity identification and functional identification

The content of the Leydig cells was estimated via histochemical staining for *3β-HSD*, as previously reported (Sadasivam et al., 2014; Yang et al., 2016). After subculture for 48 h, different concentrations (0, 5, 10, 25, 50, 75, and 100 IU/mL) of human chorionic gonadotropin (hCG) (ProSpec-Tany TechnoGene Ltd, USA) were each treated in three replicates. After 24 h of culture, the culture medium was collected, centrifuged at 1500 rpm for 5 min, and the supernatant was separated for further analysis of testosterone content.

### Cell treatment

The LPS powder (from *Escherichia coli* O111:B4, Sigma Chemicals Company, Saint Louis, MO, USA) was dissolved in phosphate-buffered saline (PBS) to prepare a high-concentration stock solution through a 0.22-µm filter, and stored at –20 °C for later use. The stock solution was subsequently diluted with medium to a final concentration of 50, 100, 200, and 400 ng/mL LPS.

The culture medium in each well of the Leydig cell culture plate was replaced with the prepared LPS, and the culture was allowed to continue. At 6, 12, 24, and 48 h, three replicate wells were set for each treatment, and the cell culture medium of each well in the 12-well culture plate was collected for measurement of testosterone, activin, and inhibin. In addition, cells were cultured for 6, 12, 24, and 48 h, and RNA was extracted and reverse transcribed into cDNA for fluorescence quantitative detection.

### Evaluation of testosterone, activin, and inhibin

Testosterone concentrations in the medium were determined by ELISA using the quantitative diagnostic kit for testosterone (Shanghai Suer Biological Technology Co. Ltd., Shanghai, China) on an ultra-microplate spectrophotometer (BioTek Instruments, Inc, Winooski, USA). Activin concentrations were also determined by ELISA using the quantitative diagnostic kit for activin (Shanghai Suer Biological Technology Co. Ltd.) on an ultra-microplate spectrophotometer (BioTek Instruments, Inc, New Jersey, USA). Similarly, inhibin concentrations were also determined by ELISA using the quantitative diagnostic kit for inhibin (Shanghai Suer Biological Technology Co. Ltd, Shanghai, China) on an ultra-microplate spectrophotometer (BioTek Instruments). The medium was diluted in the assay buffer solution provided in the kit, for which the hormone standard had been tested prior to analysis.

### Total RNA extraction and real-time PCR

Real-time quantitative PCR was performed to quantify the expression of *β-actin*, *StAR*, *3β-HSD*, *P450arom*, *FSHR*, *LHR*, and *GnIHR* mRNA in Leydig cells. Total RNA was extracted from Leydig cells using the Trizol reagent (Invitrogen, Carlsbad, California, USA) according to the manufacturer's recommendations. The RNA quality was assessed by measuring the A260:A280 ratio in the microplate spectrophotometer (BioTek, USA). Furthermore, RNA was reverse transcribed to synthesize first strand cDNA using the ReverTra Ace qRCR RT Master Mix with gDNA Remover reagent kit (Toyobo Co. Ltd., Osaka, Japan) following the manufacturer's protocol. Reverse transcription (RT) was performed at 37 °C for 15 min and terminated by heating at 50 °C for 5 min, and 98 °C for 5 min, followed by cooling at 4 °C.

Gene-specific primers were designed using the Primer 3.0 software (www.ncbi.nlm.nih.gov/tools/primer-blast/) based on the Basic Local Alignment Search Tool (BLAST), Ensemble, and GenBank databases (Table 1). Expression of β-actin mRNA was used as a reference. Real-time PCR was performed using an ABI 7500 Real-Time PCR system (Applied Biosystems, Foster City, CA, USA). Reactions were carried out using the SYBR® PrimeScriptTM RT-PCR kit (Takara Bio Inc, Japan, Takara code: DRR041A) following the manufacturer's instructions. The thermal cycling profile was as follows: 95 °C for 30 s; 40 cycles at 94 °C for 5 s; and 60 °C for 30 s. The relative expression levels of different genes in the tissues were calculated according to the 2^-ΔΔCT^ method (Livak and Schmittgen, 2001).

**Table 1:** Primer sequences used for real-time PCR.

Gene	Primer sequence (5′-3′)	Annealing temperature (°C)	Product size (bp)	Accession number
*β-Actin*	F:ATGTCGCCCTGGATTTCG R:CACAGGACTCCATACCCAAGAA	56, 57, 58	165	NM_001310421.1
*StAR*	F:GGTGGACAACGGAGACAAAG R:ATCTTGACCTCCTTGACGCT	57	165	XM_021278990.1
*3β-HSD*	F:AGAAGTGACAGGCCCAAACT R:ACATGGATCTCAGGGCACAA	58	188	XM_005028697.3
*P450arom*	F:CATCAATACCAGGGCCAGGA R:CAAGCTTGCTCCCAAATCGA	58	228	XM_021277353.1
*GnIHR*	F:CATCCTGGTGTGCTTCATCG R:ACATGGTGTTGTCAAAGGGC	56	164	XM_005028365.3
*FSHR*	F:AGCACCTTCCAAGCCTTAGA R:TGACCATGGAAGGCAGATGT	56	210	XM_021267215.1
*LHR*	F: GCTCTGTGATAACTTGCGTA R: TGAGGTTTCTGTTGTCCTTC	56	170	XM_021267245.1

### Statistical analyses

Drug treatments were performed in triplicate for the same experiments, and individual experiments were repeated at least three times. All data were presented as the mean ± standard error of mean (SEM). Statistical significance was tested using the Student’s *t*-test. *P*-values < 0.05 were considered statistically significant.

## Results

### Separation, purification, and identification of Leydig cells

**Figure 1:**
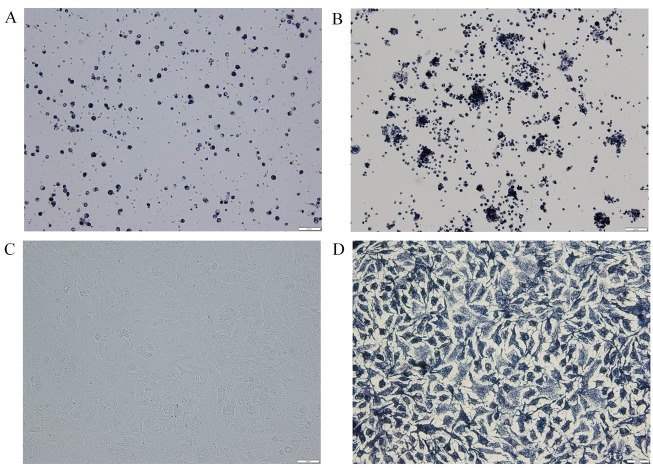
Identification of duck primary Leydig cells. (A) Identification of Leydig cells before purification; (B) Identification of Leydig cells after purification; (C) Leydig cells cultured for 72 h; (D) Identification of Leydig cells after culture for 72 h.

Duck Leydig cells were isolated from testes using collagenase digestion and then identified through 3β-HSD staining. The results showed very low purity of the Leydig cells before purification. Germ cells and cell debris were mainly present in the testes (Figure 1A). Percoll density gradient centrifugation was performed to purify the cells, and the purity of the cells was greatly improved (Figure 1B). After culture for 48 hours, we changed the medium of the Leydig cells to remove a small amount of non-adherent foreign cells or dead cells. The adherent cells grew well and had the typical characteristics of Leydig cells (Figure 1C). Further identification by 3β-HSD staining revealed that the purity of the cells exceeded 90% (Figure 1D).

**Figure 2:**
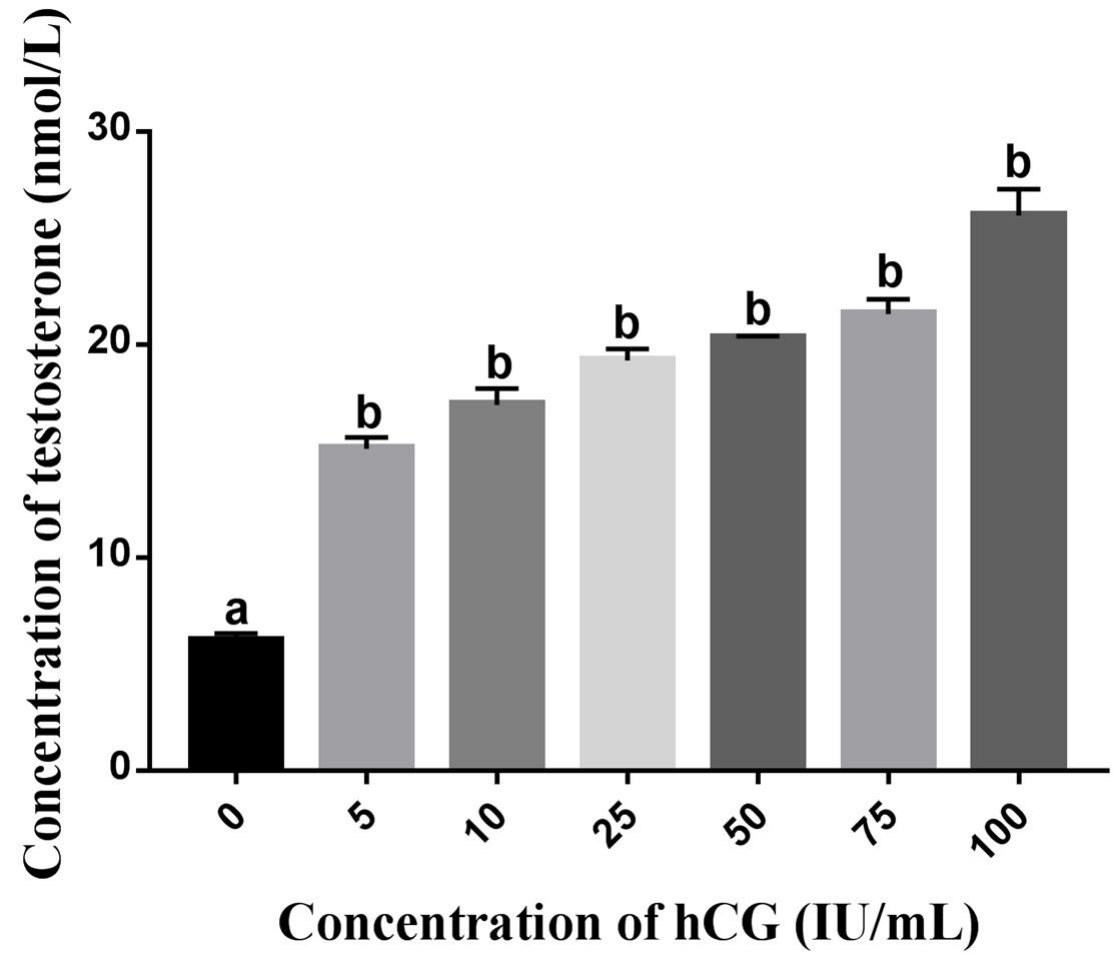
Different concentrations of hCG stimulate the secretion of testosterone in Leydig cells. Data represent mean ± SEM (n = 3); different letters indicate significant differences (*P*< 0.05).

The main function of Leydig cells is to synthesize and secrete testosterone, and they are regulated by LH or hCG (Drosdowsky et al., 1965; Keeney et al., 1988; O’Shaughnessy, 1991). Therefore, hCG was selected to stimulate the secretion of testosterone in the Leydig cells as a functional indicator. The results showed that hCG could significantly improve the secretion of testosterone in testicular stromal cells, and testosterone secretion was also upregulated with increasing hCG concentration (Figure 2).

### Effect of LPS on the secretion of hormones

**Figure 3:**
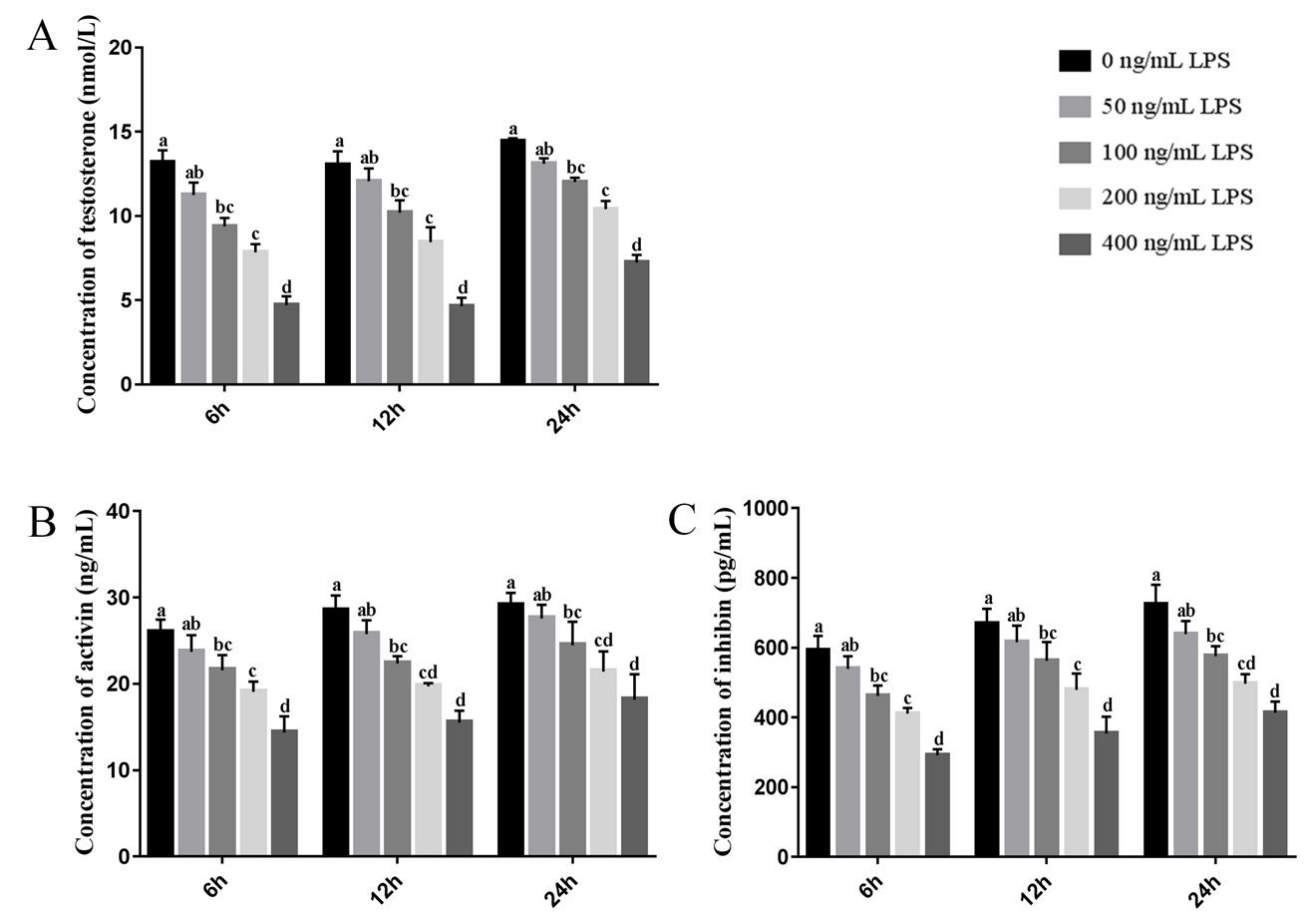
Hormone secretion in Leydig cells treated with different concentrations of LPS after 6, 12, and 24 h was analyzed. (A) Testosterone; (B) Activin; (C) Inhibin. Data represent mean ± SEM (n = 3); different letters indicate significant differences (*P*< 0.05).

The testosterone, activin, and inhibin concentrations in Leydig cells treated with 0, 50, 100, 200, and 400 ng/mL LPS were examined. Compared with the control group, the levels of testosterone, activin, and inhibin secreted in Leydig cells showed a gradual decline with increasing LPS concentration (Figure 3A, B, and C). When the concentration was above 100 ng/mL, the levels of testosterone, activin, and inhibin were all significantly reduced (*P*< 0.05) at 6, 12, and 24 h (Figure 3A, B, and C).

### Effect of LPS on gene expression

**Figure 4:**
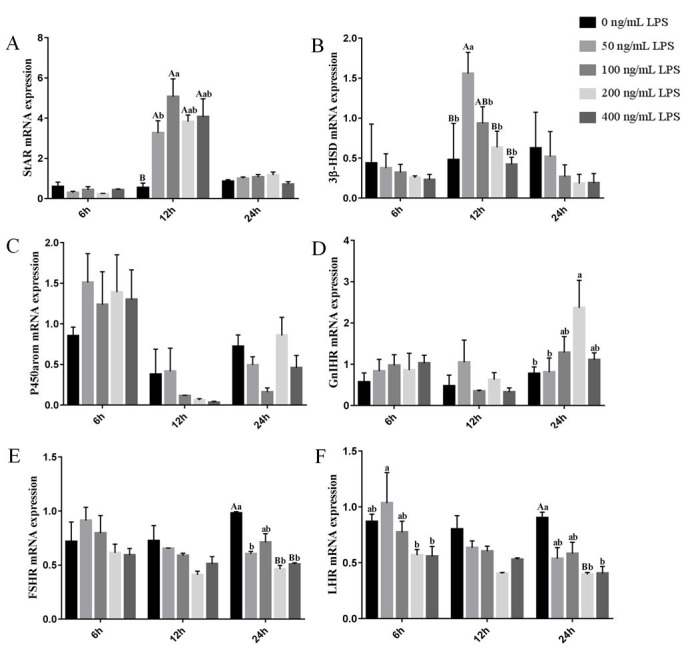
Relative expression of related genes was analyzed in Leydig cells treated with different concentrations of LPS after 6, 12, and 24 h. (A) *StAR*; (B) *3β-HSD*; (C) *P450arom*; (D) *GnIHR*; (E) *FSHR*; (F) *LHR*. Data represent mean ± SEM (n = 3); different letters on bars represent significant differences (*P*< 0.05).

The mRNA levels of *StAR*, *3β-HSD*, *P450arom*, *GnIHR*, *FSHR,* and *LHR* in Leydig cells treated with LPS were examined by qPCR. The results showed that the expression of these six genes was regulated by LPS (Figure 4). Expression of the *StAR* gene was significantly increased in Leydig cells treated with 50, 100, 200, and 400 ng/mL LPS at 12 h (*P*< 0.05), and then returned to normal levels at 24 h (Figure 4A). Expression of the *3β-HSD* gene was significantly increased in Leydig cells treated with 50 ng/mL LPS at 12 h (*P*< 0.05) (Figure 4B). Although LPS upregulated the expression of the *P450arom* gene at 6 h (*P*> 0.05), this effect was not significant (Figure 4C). Expression of the *GnIHR* gene was significantly increased in Leydig cells treated with 200 ng/mL LPS at 24 h (*P*< 0.05) (Figure 4D). Expression of the *FSHR* and *LHR* genes were both significantly reduced after treatment with LPS at 24 h (*P*< 0.05), and that of the *LHR* gene was also reduced at 6 h after treatment with 200 and 400 ng/mL LPS (Figure 4E and F).

## Discussion

The effects of hCG are similar to those of LH, which promotes testosterone secretion by the Leydig cells through interaction with the cell membrane receptor, and activates adenylate cyclase (Hedger and Eddy, 1986). Therefore, we used hCG to identify the primary cells that were isolated from the duck testicles. The results showed that the level of testosterone secreted by primary cells was significantly increased after treatment with hCG, suggesting that most of the primary cells isolated may have been Leydig cells. We also identified primary cells by 3β-HSD staining and after culture for 72 h, we found that more than 90% of the primary cells were Leydig cells. These results indicate that we successfully isolated the Leydig cells of the duck, which can be applied for further studies.

The effects of LPS include the induction of systemic inflammation and an acute stress response in the testis, leading to mitochondrial dysfunction and the activation of cell death pathways (Reddy et al., 2006; Metukuri et al., 2010). Furthermore, LPS can directly act on Leydig cells to inhibit testosterone production. Levels of serum testosterone in mice are reduced by 90% after injection with LPS (Bosmann et al., 1996; O’Bryan et al., 2000). In the primary Leydig cells isolated in the present study, the level of testosterone declined after 6 h of treatment with LPS, and the extent of this reduction became greater with increasing LPS concentration. The glycoprotein hormones, FSH and LH, are secreted by the pituitary gland and play a key role in regulating steroidogenesis (Kakar et al., 1992). Inhibin can selectively inhibit the release of FSH from cultured pituitary cells, whereas activin is a dimer of inhibin β-subunits that stimulates FSH release (McLachlan et al., 1988; Vale et al., 1988; Ying, 1988). We also detected the concentrations of activin and inhibin in the Leydig cells treated with LPS and found their changes were similar to those of testosterone. These results suggest that LPS may impair the function of Leydig cells by inhibiting their secretion of hormones.

The StAR protein is a cholesterol transport protein, mainly involved in the metabolism of cholesterol, synthetic steroid hormones. Expression of the StAR protein in mice was reduced 2 h after injection with LPS (Bosmann et al., 1996). Studies in mouse Leydig cells have demonstrated that the expressions of *StAR*, *3β-HSD*, and *P450arom* are reduced within several hours of treatment with relatively high doses of LPS (Hales et al., 1992). Interestingly, at 12 h, expression of the *StAR* gene was increased in duck Leydig cells after treatment with LPS, and *3β-HSD* mRNA levels were also increased at 12 h after treatment with low concentrations of LPS (50 and 100 ng/mL). A recent study found that the expression of *StAR* mRNA in cows was higher in cystic follicles with a high concentration of LPS than in preovulatory follicles (Shimizu et al., 2018). Low concentrations of LPS may promote the reproductive performance of animals; however, the specific mechanism needs further research.

Both FSH and LH promote steroid synthesis and testicular development, and their functions are mediated by their specific receptors FSHR and LHR (Ni et al., 2007). *GnIH* downregulated *FSH* and *LH* to regulate Leydig cells by inhibiting *GnRH* in hypothalamus or directly inhibiting LH. In this study, we found that high concentrations of LPS can increase the expression of GnIHR in Leydig cells at 24 h, and also directly suppress the expression of FSHR and LHR at 24 h. Therefore, down-regulation of *FSHR* and *LHR* gene expression may be the main cause of LPS inhibition of Leydig cells in the duck.

In summary, Leydig cells of the duck were successfully isolated and identified. We used LPS to treat Leydig cells and observed that the secretion of hormones (including testosterone, activin, and inhibin) and expression of the *FSHR* and *LHR* genes were inhibited. The results indicate that LPS can directly affect animal reproduction by impairing the function of Leydig cells. The specific regulatory mechanisms of LPS on Leydig cells need further study.

## List of abbreviations

ELISA enzyme-linked immunosorbent assay

FBS fetal Bovine Serum

FSH follicle stimulating hormone

FSHR follicle stimulating hormone receptor

GnIH gonadotropin-inhibitory hormone

GnIHR gonadotropin-inhibitory hormone receptor

GnRH gonadotropin-releasing hormone

LH luteinizing hormone

LHR luteinizing hormone receptor

LPS lipopolysaccharide

PBS phosphate buffered saline

RT reverse transcription

SEM standard error of mean

StAR steroidogenic acute regulatory
